# Prognostic value of GRACE and EDACS scores in non-STEMI patients presenting to the emergency department

**DOI:** 10.1515/med-2026-1497

**Published:** 2026-08-14

**Authors:** Omer Faruk Cakiroglu, Julide Gurbuz, Omer Faruk Oz, Yilmaz Ersoz, Bahadir Taslidere, Basar Cander

**Affiliations:** Department of Emergency Medicine, Kartal Doktor Lutfi Kirdar City Hospital, Istanbul, Türkiye; Department of Emergency Medicine, Bezmialem Vakıf University, School of Medicine, Istanbul, Türkiye; Department of Emergency Medicine, Sancaktape Sehit Ilhan Varank Training and Research Hospital, Istanbul, Türkiye

**Keywords:** NSTEMI, GRACE score, EDACS score

## Abstract

**Objectives:**

This study primarily aimed to evaluate the association of EDACS and GRACE scores with angiographically significant coronary lesions in patients presenting with NSTEMI. The secondary objective was to assess their relationship with 30-day mortality.

**Methods:**

This retrospective single-center study included adults diagnosed with NSTEMI between January 2022 and December 2024. Demographic and clinical characteristics, GRACE and EDACS scores, angiographic findings, and 30-day mortality were obtained from electronic hospital records. Significant coronary lesions were defined as ≥70 % stenosis or the need for stent placement. Statistical analyses included Mann–Whitney U, Chi-square, logistic regression, and ROC analyses.

**Results:**

Among 332 patients, both GRACE and EDACS scores were significantly higher in non-survivors. GRACE high-risk classification was strongly associated with mortality, whilst EDACS (≥16) showed a non-significant trend. EDACS demonstrated superior discriminatory ability for significant coronary lesions, whereas GRACE was more closely associated with mortality risk. An EDACS cut-off >12.5 yielded 87.7 % sensitivity and 40.0 % specificity. Logistic regression identified male sex, hypercholesterolaemia, EDACS ≥16, and systolic blood pressure as independent predictors of significant lesions.

**Conclusions:**

GRACE predicted mortality, whereas EDACS better identified significant coronary lesions; combined use showed marginal improvement in model fit but did not significantly enhance discriminatory performance.

## Introduction

Non-ST-Elevation Myocardial Infarction (NSTEMI) is a condition of subendocardial ischemia that develops due to partial coronary occlusion caused by atherosclerotic plaque rupture or erosion [1]. The one-year mortality rate is 10–15 % [2]. Age, diabetes, hypertension, dyslipidemia, smoking, obesity, and chronic kidney disease are the main determinants of poor prognosis; in advanced age and in women, atypical symptoms may lead to diagnostic delay and increased mortality [3]. Due to its heterogeneous course, early and accurate risk stratification is critically important [4]. The Global Registry of Acute Coronary Events (GRACE) score is the gold standard for mortality prediction and includes age, vital signs, Killip class, and troponin [5]; however, the requirement for multiple parameters and the high weighting of age may create practical limitations [6], 7]. Emergency Department Assessment of Chest Score (EDACS), developed for rapid assessment in the emergency department, is based on age, sex, risk factors, and symptoms, and is effective in identifying low-risk cases [8].

Therefore, rather than directly validating EDACS against GRACE for identical clinical outcomes, this study aimed to explore whether EDACS, originally developed as a discharge-oriented chest pain risk stratification tool, may also reflect anatomical coronary risk in patients with NSTEMI. Accordingly, the present study investigated the relationship between EDACS classification and angiographically significant coronary stenosis in NSTEMI patients. Since angiographically significant coronary stenosis is associated with adverse cardiovascular outcomes in acute coronary syndromes, evaluating the relationship between EDACS classification and coronary anatomy may provide clinically relevant complementary information. To provide prognostic context, EDACS findings were evaluated alongside the GRACE score, which represents a mortality-oriented risk assessment framework in NSTEMI.

## Materials and methods

In this retrospective, single-center study, we included adult patients (≥18 years) diagnosed with NSTEMI in the emergency department from January 1, 2022, through December 31, 2024. The diagnosis of non-STEMI was established according to the 2023 ESC guidelines [9]. NSTEMI was defined as the presence of clinical symptoms suggestive of myocardial ischemia and/or electrocardiographic findings compatible with acute coronary syndrome, together with elevated cardiac troponin levels above the 99th percentile upper reference limit, in the absence of persistent ST-segment elevation. Patients were identified using ICD-10 codes and subsequently confirmed by reviewing emergency department records, laboratory results, electrocardiographic findings, cardiology consultation notes, and coronary angiography reports. Patients younger than 18 years, those diagnosed with ST-elevation myocardial infarction, unstable angina without troponin elevation, missing clinical or laboratory data required to calculate GRACE or EDACS scores, unavailable angiographic records, and repeated admissions during the study period were excluded from the analysis. Patient data were obtained from the hospital system using ICD-10 codes, emergency department patient identification forms, and primary percutaneous coronary angiography images. Killip classification was evaluated based on clinical findings at presentation and recorded in the emergency department patient identification forms. Thirty-day all-cause mortality was defined as death occurring within 30 days after the index emergency department presentation. Follow-up data were obtained from the hospital electronic medical record system, including inpatient records, discharge summaries, outpatient follow-up visits, and mortality records available within the hospital database. Mortality during the index hospitalization and after discharge within the 30-day period was included in the analysis. GRACE and EDACS scores were calculated for all included patients using admission clinical and laboratory parameters. The tertiles of risk categories used were as follows: for in-hospital mortality, low risk for GRACE score ≤108, intermediate risk for GRACE score between 109 and 140, and high risk for GRACE score ≥141 [10]. EDACS was primarily analyzed using the predefined high-risk threshold of ≥16, while ROC-derived cut-off values were explored in secondary analyses [11]. The performance of percutaneous coronary intervention was evaluated separately as a treatment-related outcome. Similar classifications have been used in previous studies [12], 13]. Significant coronary lesions were defined as ≥70 % luminal stenosis, in line with commonly accepted angiographic thresholds used in prior studies evaluating clinically relevant obstructive coronary artery disease [14]. The primary endpoint of the study was the presence of angiographically significant coronary stenosis, defined as ≥70 % luminal narrowing on coronary angiography. The secondary endpoint was 30-day all-cause mortality following the index emergency department presentation. Coronary angiography and revascularization decisions were performed according to the treating cardiology team’s clinical judgment and current guideline recommendations. The presence of angiographically significant coronary stenosis and PCI findings were included in the analysis as clinically relevant anatomical outcomes.

In the statistical power analysis, assuming a medium effect size (Cohen’s f=0.25), a 5 % significance level (α=0.05), 80 % power (1-β=0.80), 95 % confidence interval, and 5 % margin of error, with a standard deviation of 0.5 % and Z-score of 1.96, the minimum sample size required to detect a difference between two groups was calculated as 298.

Data analysis was performed using IBM SPSS V25. Categorical variables were presented as frequencies and percentages. The normality of quantitative data was assessed using the Shapiro–Wilk test. Since normal distribution assumptions were not met, the Mann–Whitney U test was used for comparisons between quantitative variables and dichotomous categorical variables. Comparisons between two categorical variables were performed using the Chi-square test.

To identify factors affecting survival and the decision for primary percutaneous coronary intervention, multivariate binary logistic regression analysis was performed using variables that were significant in univariate analyses.

The diagnostic performance of GRACE and EDACS scores for predicting angiographically significant coronary lesions was evaluated using ROC curve analysis. A type I error rate of 0.05 was considered acceptable. Additionally, a combined logistic regression model incorporating both GRACE and EDACS scores as continuous variables was constructed to evaluate their joint discriminatory performance. Predicted probabilities derived from this model were used for ROC curve analysis, and model discrimination was compared using the likelihood ratio test and the DeLong method. This study was performed in accordance with the principles of the Declaration of Helsinki (as revised in 2013). The Ethics Committee of Bezmialem Vakıf University approved the study (decision date: 14 May 2025; reference number: 2025/107). Informed consent was waived because of the retrospective design of the study.

## Results

A total of 332 patients diagnosed with NSTEMI in the emergency department were included. Overall 30-day mortality was 5.4 % (n=18). Non-survivors were significantly older than survivors (median 72 vs. 63 years; p=0.008). No significant between-group differences were observed for sex, comorbidities, hypertension, diabetes mellitus, or ST-segment deviation. Hypercholesterolemia was associated with markedly lower mortality (0.8 vs. 8.0 %; p=0.005), and cardiac arrest at presentation was the strongest predictor, occurring in 50 % of non-survivors vs. 4.6 % of survivors (p<0.001). Mortality increased progressively with Killip class, from 3.3 % in Class I to 50 % in Class IV (p=0.011; Table 1).

**Table 1:** Comparison of demographic and clinical characteristics according to mortality.

		Survivor median (min−max)	Non-survivor median (min−max)	Z	p-Value
Age		63 (18–94)	72 (22–96)	−2.632	0.008^a^

		**Survivor n (%)**	**Non-survivor n (%)**	**Chi-square**	**p-Value**

Sex	Female	98 (94.2)	6 (5.8)	0.036	0.850
	Male	216 (94.7)	12 (5.3)		

**Chronic disease**					

Absent		42 (95.5)	2 (4.5)	0.076	0.783
Present		272 (94.4)	16 (5.6)		

**Hypertension**					

Absent		94 (94)	6 (6)	0.093	0.760
Present		220 (94.8)	12 (5.2)		

**Diabet**e**s mellitus**					

Absent		181 (95.8)	8 (4.2)	1.210	0.271
Present		133 (93)	10 (7)		

**Hyperlipidemia**					

Absent		195 (92)	17 (8)	7.716	0.005^a^
Present		119 (99.2)	1 (0.8)		

**Cardiac arrest on arrival**					

Absent		311 (95.4)	15 (4.6)	23.681	<0.001^a^
Present		3 (50)	3 (50)		

**ST-segment deviation**					

Absent		217 (96)	9 (4)	2.860	0.091
Present		97 (91.5)	9 (8.5)		

**Killip classification**					

Class I		232 (96.7)	8 (3.3)	11.116	0.011^a^
Class II		50 (89.3)	6 (10.7)		
Class III		31 (91.2)	3 (8.8)		
Class IV		1 (50)	1 (50)		

**Diaphoresis**					

Absent		223 (95.3)	11 (4.7)	0.803	0.370
Present		91 (92.9)	7 (7.1)		

**Radiating pain to extremities**					

Absent		55 (90.2)	6 (9.8)	2.840	0.092
Present		259 (95.6)	12 (4.4)		

**Pain change to respiration**					

Absent		276 (94.2)	17 (5.8)	0.704	0.402
Present		38 (97.4)	1 (2.6)		

**Pain change to palpation**					

Absent		245 (94.2)	15 (5.8)	0.282	0.595
Present		69 (95.8)	3 (4.2)		

Superscript “a” indicates a statistically significant p-value. Statistical significance was defined as p<0.05.

Both GRACE and EDACS scores were significantly higher in non-survivors. Median GRACE scores were 140.5 (range 74–214) in non-survivors vs. 97 (range 38–208) in survivors (p<0.001). Median EDACS scores were 21 (range 7–28) in non-survivors vs. 17.5 (range 1–34) in survivors (p=0.022). When stratified by GRACE risk category, mortality increased from 2 % in the low-risk group (≤108) to 16.1 % in the high-risk group (≥141; p<0.001). The EDACS high-risk group (≥16) demonstrated a numerically higher but statistically non-significant mortality rate compared with the low-risk group (7.0 vs. 2.6 %; p=0.090; Table 2). Mortality did not differ significantly between patients with and without angiographically significant coronary lesions (6.3 vs. 2.5 %; p=0.185). A significant association was observed between GRACE risk categories and EDACS risk classification (χ^2^=27.842; p<0.001). Among patients in the low-risk GRACE category (≤108), 79.5 % were classified as low risk by EDACS (<16), whereas 22.3 % of patients in the high-risk GRACE group (≥141) were classified as high risk by EDACS (≥16; Table 3).

**Table 2:** Comparison of scoring systems and clinical outcomes according to mortality.

	Survivors median (min−max)	Non-survivors median (min−max)	Z-score	p-Value
GRACE score	97 (38–208)	140.5 (74–214)	−3.78	<0.001^a^
EDACS score	17.5 (1–34)	21 (7–28)	−2.293	0.022^a^

**GRACE score in-hospital mortality risk categories**				

≤108	197 (98)	4 (2)	14.845	<0.001^a^
109–140	70 (93.3)	5 (6.7)		
≥141	47 (83.9)	9 (16.1)		

**EDACS risk categories**				

<16	114 (97.4)	3 (2.6)	2.877	0.090
≥16	200 (93)	15 (7)		

**Angiographically significant coronary lesions**				

No significant coronary lesion	78 (97.5)	2 (2.5)	1.755	0.185
Significant coronary lesion	236 (93.7)	16 (6.3)		

Superscript “a” indicates a statistically significant p-value. Statistical significance was defined as p<0.05.

**Table 3:** Comparisons between EDACS and GRACE scores.

	EDACS			
GRACE	<16	≥16	Chi-square	p-Value
≤108	93 (79.5)	108 (50.2)	27.842	<0.001^a^
109–140	16 (13.7)	59 (27.4)		
≥141	8 (6.8)	48 (22.3)		

Superscript “a” indicates a statistically significant p-value. Statistical significance was defined as p<0.05.

**Figure 1:**
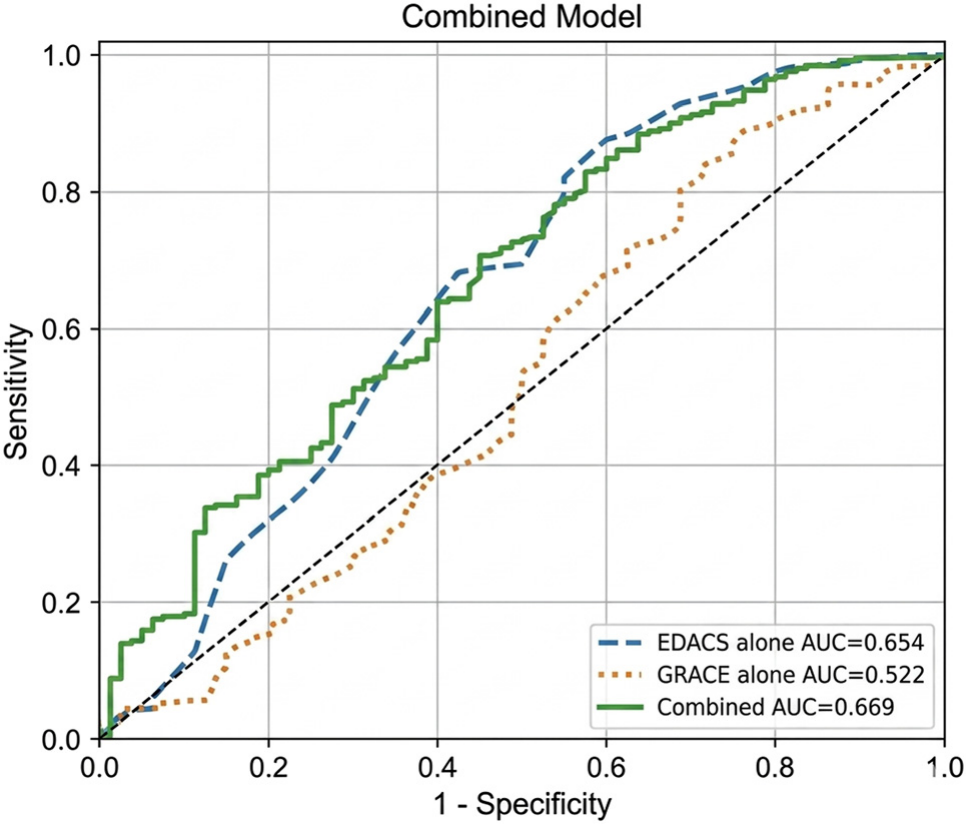
ROC curves of EDACS, GRACE, and combined EDACS-GRACE models for predicting angiographically significant coronary lesions.

ROC analysis showed that EDACS had statistically significant discriminatory ability for predicting angiographically significant coronary lesions (AUC: 0.654, 95 % CI: 0.578–0.730; p<0.001), whereas GRACE demonstrated no statistically significant discriminatory value for this endpoint (AUC: 0.522, 95 % CI: 0.443–0.600; p=0.560). A combined model incorporating both EDACS and GRACE demonstrated a modest increase in discriminatory performance (AUC: 0.669), although the overall performance remained below the conventional threshold considered acceptable for stand-alone clinical decision-making. The Youden-index-derived EDACS cut-off of >12.5 yielded a sensitivity of 0.877 and a specificity of 0.400 and was considered exploratory; the predefined threshold of ≥16 was retained for categorical analyses (Table 4, Figure 1).

**Table 4:** ROC analysis of GRACE, EDACS, and combined EDACS-GRACE models for predicting angiographically significant coronary lesions.

	AUC	95 % CI	SE	p-Value	Cut-off	Sens	Spes
GRACE	0.522	0.443–0.600	0.040	0.560	>67	0.889	0.237
EDACS	0.654	0.578–0.730	0.039	<0.001^a^	>12.5	0.877	0.400
Combined	0.669	0.595–0.743	0.038	<0.001^a^	Prob >0.751	0.706	0.550

Superscript “a” indicates a statistically significant p-value. Statistical significance was defined as p<0.05.

Patients classified as high risk by EDACS (≥16) had a significantly higher prevalence of angiographically significant coronary lesions compared with the low-risk group (81.9 vs. 65.0 %; p=0.001). Consistently, median EDACS scores were significantly higher in patients with significant lesions (19 vs. 15.5; p<0.001). In contrast, GRACE categories and continuous GRACE scores were not significantly associated with angiographic findings (Table 5).

**Table 5:** Comparison of patients’ scoring systems and clinical outcomes according to PCI findings.

GRACE in-hospital mortality risk categories	No significant coronary lesion, n (%)	Significant coronary lesion, n (%)	Chi-square	p-Value
≤108	47 (23.4)	154 (76.6)	0.753	0.686
109–140	17 (22.7)	58 (77.3)		
≥141	16 (28.6)	40 (71.4)		

**EDACS high-risk cutoff (16 points)**				

<16	41 (35)	76 (65)	11.836	0.001^a^
≥16	39 (18.1)	176 (81.9)		

	**No significant coronary lesion median (min−max)**	**Significant coronary lesion median (min−max)**	**Z-score**	**p-Value**

GRACE score	97.5 (42–182)	99 (38–214)	−0.583	0.560
EDACS score	15.5 (1–34)	19 (3–32)	−4.159	<0.001^a^

Superscript “a” indicates a statistically significant p-value. Statistical significance was defined as p<0.05.

In multivariable logistic regression, male sex (OR: 2.184; 95 % CI: 1.257–3.794; p=0.006), hypercholesterolemia (OR: 1.839; 95 % CI: 1.031–3.281; p=0.039), high-risk EDACS classification (≥16; OR: 2.07; 95 % CI: 1.207–3.551; p=0.008), and systolic blood pressure (OR: 1.009; 95 % CI: 1.002–1.017; p=0.015) were independently associated with the presence of angiographically significant coronary lesions (Table 6). In the combined logistic regression model, EDACS remained a strong independent predictor (OR: 1.140; 95 % CI: 1.082–1.200; p<0.001), while GRACE demonstrated a significant inverse association consistent with a suppressor effect (OR: 0.991; 95 % CI: 0.983–0.999; p=0.034). Although the combined model achieved a significantly better fit than EDACS alone by likelihood ratio test (χ^2^=4.488; df=1; p=0.034), pairwise DeLong comparison did not confirm a significant improvement in AUC (Z=0.288; p=0.773) (Table 7).

**Table 6:** Logistic regression analysis for PCI findings.

Variable	B	SE	Wald	p-Value	OR (95 % CI)
Sex (ref: female)	0.781	0.282	7.677	0.006^a^	2.184 (1.257–3.794)
Cholesterol (ref: absent)	0.609	0.295	4.256	0.039^a^	1.839 (1.031–3.281)
EDACS high risk ≥16 (ref: absent)	0.728	0.275	6.982	0.008^a^	2.07 (1.207–3.551)
Systolic blood pressure	0.009	0.004	5.921	0.015^a^	1.009 (1.002–1.017)
Constant	−0.39	0.616	0.402	0.526	0.677 (−)

Superscript “a” indicates a statistically significant p-value. Statistical significance was defined as p<0.05.

**Table 7:** Combined logistic regression model (EDACS + GRACE) and pairwise comparison of discriminatory performance for angiographically significant coronary lesions.

Table 7A-combined logistic regression model					
Variable	B	SE	Wald	p-Value	OR (95 % CI)
GRACE score	−0.009	0.004	4.485	0.034^a^	0.991 (0.983–0.999)
EDACS score	0.131	0.026	24.661	<0.001^a^	1.140 (1.082–1.200)

**Table 7B-pairwise model comparison**					

**Comparison**	**ΔAUC**	**LRT χ** ^**2**^ **(df=1)**	**LRT p**	**DeLong Z**	**DeLong p**

Combined vs. EDACS alone	+0.015	4.488	0.034^a^	0.288	0.773
Combined vs. GRACE alone	+0.147	26.853	<0.001^a^	–	–

^a^p<0.05. ΔAUC, difference in area under the curve; LRT, likelihood ratio test; DeLong, pairwise ROC curve comparison method; –, not applicable. Superscript “a” indicates a statistically significant p-value. Statistical significance was defined as p<0.05.

## Discussion

In this study, EDACS demonstrated a significant association with angiographically significant coronary lesions in NSTEMI patients, whereas GRACE showed limited discriminatory value for this anatomical endpoint, suggesting that the two scores may reflect different dimensions of clinical risk assessment. Risk stratification in NSTEMI remains a central component of clinical decision-making, requiring an understanding not only of individual scoring systems but also of how they relate to and complement each other. The data obtained in our study revealed a statistically significant and strong relationship between EDACS and GRACE scores (p<0.001). The concordance between low EDACS scores and lower GRACE categories suggests consistency in identifying clinically stable patients, whereas the limited overlap in higher categories indicates variability in classifying those at elevated risk. This suggests that EDACS may have limited sensitivity in identifying high-risk cases.

In the study by Santos et al., the GRACE score was reported to be a significant and strong prognostic indicator for predicting short-term major cardiac events in patients with NSTEMI [15]. In the study by Chowdhury et al., the GRACE score was found to be a more reliable and powerful prognostic tool for identifying high-risk patients with NSTEMI [16].

Multiple studies, particularly the meta-analysis by D’Ascenzo et al., and the findings of Correira et al., demonstrated that the GRACE score is the most reliable and recommended risk score for short- and long-term prognosis assessment, predicting severe coronary artery disease and adverse clinical outcomes in patients with NSTEMI [17], 18]. In the study by Akbaş et al., EDACS was shown to be an effective tool for safely discharging low-risk patients presenting to the emergency department with chest pain [19].

Chapman et al. demonstrated that EDACS-based pathways combined with serial high-sensitivity troponin testing achieved high negative predictive value for 30-day myocardial infarction or cardiac death in patients with suspected acute coronary syndrome, while GRACE remained primarily a prognostic framework focused on mortality risk [20]. These findings support the concept that EDACS may reflect different clinical dimensions of risk assessment compared with traditional mortality-oriented models, particularly in the identification of lower-risk patients.

In the study by Hasballa et al., EDACS demonstrated limited accuracy (42.2 %) in predicting MACE in high-risk patients, suggesting that its use in this population may not be appropriate [21]. In the study by Than et al., EDACS was calculated in 279 patients, and 41.6 % were classified as low risk; none of these low-risk patients developed MACE within 30 days [8]. In another study by Meier et al. involving 4,551 patients, the sensitivity of EDACS for ruling out 30-day MACE in low-risk patients was found to be 72 %, and its AUC for predicting 30-day MACE was 0.74, performing inferior to HEATRT, GRACE, Thrombolysis In Myocardial Infarction (TIMI), and T-MAC scores [22].

Given this information, the GRACE score – validated in numerous studies remains the gold standard in patients with NSTEMI for assessing short- and long-term prognosis.

GRACE provides a more comprehensive prognostic assessment by incorporating multiple clinical and laboratory parameters, whereas EDACS offers a simpler, easier-to-apply, and time-efficient risk score. Therefore, EDACS may be effective in safely ruling out low-risk patients, but may not be as robust as GRACE in identifying high-risk cases.

In our study, the relationship between EDACS score and mortality was evaluated and demonstrated limited performance in identifying high-risk patients, and its prognostic value may need confirmation in larger, more comprehensive future studies.

Although EDACS was originally designed for rapid emergency department triage and safe discharge assessment, several of its components, including age, sex, cardiovascular risk factors, and symptom characteristics, are closely associated with underlying atherosclerotic burden. In this context, evaluating the relationship between EDACS classification and angiographically significant coronary stenosis may provide additional insight into anatomical coronary risk in NSTEMI patients. From a clinical perspective, the EDACS score demonstrated a notable sensitivity of 87.7 % at a threshold of >12.5. This high sensitivity is essential in the emergency department, where the priority is to establish a ‘safe screening’ strategy to avoid missing high-risk patients. By identifying nearly 88 % of those with significant coronary stenosis, EDACS serves as a valuable diagnostic aid. It effectively reduces the risk of overlooking critical anatomical findings, particularly in instances where traditional hemodynamic scores like GRACE fail to provide predictive value for such lesions. The ROC-derived cut-off (>12.5) was considered exploratory, while the predefined threshold of ≥16 was retained for categorical analyses. Given the low specificity at the ROC-derived cut-off, EDACS should not be considered a standalone tool for guiding invasive strategies. However, the relatively low specificity and resulting high false-positive rate substantially limit the utility of EDACS as a stand-alone tool for anatomical risk prediction. In the present study, EDACS was evaluated for its association with angiographically significant coronary lesions rather than for guiding revascularization decisions, which are influenced by multiple clinical and procedural factors. A critical divergence emerged in our analysis: while the GRACE score remains the benchmark for hemodynamic risk and mortality, EDACS more accurately reflects anatomical risk, specifically significant coronary stenosis. This distinction likely stems from their differing architectures; GRACE is weighted toward acute clinical instability and age, whereas EDACS integrates symptom characteristics and cardiovascular risk factors. As these parameters are more directly linked to the underlying atherosclerotic burden, EDACS may serve as a more effective surrogate for predicting high-grade coronary narrowing than the mortality-focused metrics of the GRACE score. Therefore, EDACS may serve as a complementary screening aid in identifying patients who could benefit from anatomical risk assessment via invasive coronary angiography. This application appears particularly relevant in cases where conventional laboratory and hemodynamic markers do not yet indicate advanced pathology, potentially offering an additional layer of risk stratification in the emergency setting. However, due to low specificity, results should be interpreted alongside with other clinical parameters.

The observed association between higher EDACS values and significant coronary lesions should be interpreted with caution. EDACS is a composite score that incorporates prior coronary artery disease and symptom characteristics, which may inherently increase its apparent association with angiographic findings. In our multivariable model, an EDACS score ≥16 was identified as an independent predictor of significant coronary lesions (OR: 2.07; 95 % CI: 1.207–3.551; p=0.008). Exploratory combined ROC analysis showed a modest improvement in discriminatory performance when EDACS and GRACE were integrated. However, the combined model still demonstrated an AUC below 0.70, suggesting limited incremental clinical utility.

These findings suggest that EDACS and GRACE may reflect different clinical dimensions of risk assessment in NSTEMI, including anatomical and prognostic risk. Further prospective validation studies are required before any potential combined clinical application can be considered. Rather than replacing established prognostic tools, EDACS may serve as a complementary clinical aid, particularly in the initial risk stratification of lower-risk patients, while GRACE remains the reference standard for prognostic assessment in NSTEMI. In multivariable analysis, male sex, hypercholesterolemia, higher EDACS category, and systolic blood pressure were associated with angiographically significant coronary lesions. As EDACS is a composite clinical score, its inclusion in regression models should be interpreted as exploratory rather than as independent validation of predictive performance.

GRACE and EDACS were originally developed for different clinical purposes. While GRACE is a prognostic score designed to estimate short- and long-term mortality risk in patients with acute coronary syndromes, EDACS was developed as a rapid risk stratification tool for patients presenting with undifferentiated chest pain in the emergency department. Therefore, the present analysis should not be interpreted as a direct head-to-head validation of these scores for identical endpoints, but rather as an exploratory assessment of their performance within a diagnosed NSTEMI population.

### Limitations

This study has several limitations. First, its retrospective, single-center design may limit the generalizability of the findings. Second, while the number of mortality events was consistent with existing literature, the small sample size for this endpoint suggests that mortality-related results should be interpreted with caution. Third, both GRACE and EDACS demonstrated AUC values below the conventional 0.70 threshold for strong discrimination for the outcomes evaluated in this study. Although the combined EDACS-GRACE model demonstrated slightly improved discrimination compared with either score alone, the overall AUC remained below the conventional threshold for robust clinical applicability. Therefore, these findings should be interpreted as exploratory and hypothesis-generating rather than practice-changing. Detailed treatment-related variables such as antiplatelet regimens, anticoagulant therapy, and timing of revascularization were not consistently available in this retrospective dataset and therefore could not be comprehensively analyzed. These unmeasured treatment-related factors may have influenced clinical outcomes and introduced residual confounding. While this represents a statistical limitation in predictive power, these results should be viewed as exploratory findings that contribute to the literature by highlighting a novel application of EDACS in anatomical risk assessment. Finally, the reliance on patient self-reports for certain clinical variables may introduce potential information bias.

## Conclusions

In this exploratory retrospective study, EDACS demonstrated an association with angiographically significant coronary lesions, whereas GRACE remained more strongly associated with mortality risk in patients with NSTEMI. These findings suggest that the two scores may reflect different aspects of clinical risk assessment rather than interchangeable prognostic functions. Further prospective multicenter studies are required to validate the potential role of EDACS in anatomical risk stratification.
